# Supplementary material for: Deep reptilian evolutionary roots of a major avian respiratory adaptation
Source: Commun Biol. 2023 Jan 17;6:3. doi: 10.1038/s42003-022-04301-z (PMC9845227; doi:10.1038/s42003-022-04301-z)
Supplement: Supplementary file 2 — Supplementary Information [file 42003_2022_4301_MOESM2_ESM.docx]

**Supplementary Information to Deep reptilian evolutionary roots of a major avian respiratory adaptation**

Yan-yin Wang^1^, Leon P.A.M. Claessens^2^, Corwin Sullivan^1,3^

^1^Department of Biological Sciences, CW 405 Biological Sciences Building, University of Alberta, Edmonton, AB, T6G 2E9, Canada

^2^Maastricht Science Programme, Faculty of Science and Engineering, Maastricht University, Maastricht, The Netherlands

^3^Philip J. Currie Dinosaur Museum, Wembley, AB, T0H 3S0, Canada

Corresponding Author: Yan-yin Wang

Email: yanyin@ualberta.ca

Supplementary information includes:

1. list of the archosaur specimens with uncinate processes and/or uncinate scars examined in this study
2. discussion of the implausibility of alternate interpretations of the features we identify as uncinate scars
3. detailed description of the procedures used for the ancestral state reconstruction
4. list of character codings used in the ancestral state reconstruction
5. list of phylogenetic studies used to compile the informal supertree
6. list of reference for the first and last appearance data of taxa used in the ancestral state reconstruction
7. probabilities of the three ancestral states in selected archosaur clades

**Table 1. Specimens with uncinate processes and/or uncinate scars examined in this study**

| Taxon | Specimen number | Morphology of uncinate processes and/or scars | Number of ribs | Proximal ridge |
| --- | --- | --- | --- | --- |
| *Caiman crocodilus* | ROM R6872 | U: tab-like processes  US: narrow, irregular strip | 1 | A |
| *Caiman crocodilus* | ROM R7077 | U: tab-like processes  US: narrow, irregular strip | 5 | A |
| Crocodylia indet. | AMNH 7900 | US: narrow strip | 1 | A |
| *Araripesuchus gomesii* | AMNH 24450 | U: hook-like processes | 7 | A |
| Aetosauria indet. | NMMNH P50048 | US: suboval concavities | 1 | A |
| Phytosauria indet. | YPM 6649 | US: suboval concavities | 2 | A |
| Phytosauria indet. | NMMNH P60401 | US: elongate groove | 1 | A |
| *Rhea americana* | UAMZ 5019 | US: suboval concavities | 6 | A |
| *Rhea* sp. |  | U: hook-like processes  US: suboval concavity | 6 with U  1 with US | A |
| *Casuarius australis* | UAMZ 1369 | U: plate-like processes  US: suboval concavities | 3 iso U  6 with US | A |
| *Ardea herodias* | UAMZ4048 | U: slender processes  US: suboval concavities | 6 with U  3 with US | A |
| *Bubo virginianus* | UAMZ 6846 | U: hook-like processes | 2 with U  5 iso U  7 with US | A |
| *Gavia immer* | UAMZ 1793 | U: hook-like processes  US: suboval concavities | 3 with U  9 iso U  8 with US | A |
| *Saurornitholestes langstoni* | UALVP 55700 | U: hook-like processes | 5 | A |
| *Saurornitholestes langstoni* | TMP 88.121.39 | US: suboval concavities | 2 | A |
| *Linheraptor exquisitus* | IVPP V 16923 | US: hook-like processes | 4 | A |
| *Struthiomimus altus* | AMNH 5355 | US: suboval concavity | 1 | P |
| *Daspletosaurus torosus* | CMN 8506 | US: suboval concavities | 10 | P |
| *Gorgosaurus libratus* | UALVP 10 | US: suboval concavities | 6 | P |
| *Albertosaurus sarcophagus* | TMP 99.50.41 | US: irregularly shaped concavity | 1 | A |
| *Albertosaurus sarcophagus* | TMP 99.50.42 | US: irregularly shaped concavity | 1 | A |
| *Allosaurus fragilis* | AMNH 5753 | US: protruding boss | 1 | A |
| Tyrannosauridae indet. | TMP 81.16.285 | US: suboval concavity | 1 | P |
| Tyrannosauridae indet. | TMP 86.16.285 | US: suboval concavity | 1 | P |
| Tyrannosauridae indet. | TMP 92.36.1231 | US: suboval concavity | 1 | P |
| Tyrannosauridae indet. | TMP 94.12.960 | US: suboval concavity | 1 | P |
| *Apatosaurus excelsus* | YPM 1981 | US: suboval concavities | 2 | A |
| *Gryposaurus notabilis** | AMNH 5350 | US: slender strips | 2 | A |
| *Gryposaurus latidens #* | AMNH 5465 | US: slender strip | 1 | A |
| *Bactrosaurus johnsoni* | AMNH 6553 | US: expanded suboval concavity | 1 | A |
| *Tenontosaurus tilletti* | AMNH 3040 | US: suboval concavities | 2 | A |
| *Zephyrosaurus schaffi* | MCZ 4392 | US: slender strip | 1 | A |
| *Parksosaurus warreni* | ROM 804 | U: tab-like processes | 3 | A |
| Hadrosauridae indet. | AMNH 5896 | US: slender strip | 1 | A |
| Hadrosauridae indet. | TMP 82.13.15 | US: slender strip | 1 | A |
| *Centrosaurus* sp. | TMP 82.18.16 | US: slender strip | 1 | A |
| *Centrosaurus* sp. | TMP 82.19.41 | US: slender strip | 1 | A |
| *Centrosaurus* sp. | TMP 82.18.56 | US: slender strip | 1 | A |
| *Centrosaurus* sp. | TMP 82.18.281 | US: slender strip | 1 | A |
| *Centrosaurus* sp. | TMP 96.176.135 | US: slender strip | 1 | A |
| *Centrosaurus* sp. | ROM 767 | US: slender strips | 2 | A |
| *Pachyrhinosaurus lakustai* | UALVP 57289 | US: slender strip | 1 | A |
| *Leptoceratops gracilis* | CMN 8889 | US: suboval concavities | 2 | A |
| Ceratopsidae indet. | AMNH 5422 | US: slender strip | 1 | A |
| Ceratopsia indet. | NMMNH P22797 | US: slender strip | 1 | A |
| *Panoplosaurus mirus* | ROM 1215 | US: expanded, suboval concavities | 2 | A |
| *Edmontonia longiceps* | CMN 8531 | US: expanded, suboval concavities | 3 | A |
| *Sauropelta edwardsi* | AMNH 3032 | US: expanded, suboval concavity | 1 | A |
| *Euplocephalus tutus* | AMNH 5337 | US: expanded, suboval concavities | 2 | A |
| *Stegosaurus stenops* | YPM 1856 | US: suboval concavity | 1 | A |
| *Stegosaurus stenops* | AMNH 650 | US: suboval concavity | 1 | A |
| *Stegosaurus* sp. | AMNH 5752 | US: suboval concavity | 1 | A |
| Dinosauria indet. | NMMNH P50406 | US: suboval concavity | 1 | A |

Abbreviation: A, absent; iso, isolated; P, present; U, uncinate process; US, uncinate scar.

**Implausibility of alternate interpretations of the uncinate scars**

Possible alternate interpretations of the features we identify as uncinate scars warrant further discussion. Here we exclude muscle scars and bite marks as plausible identifications for these features.

The uncinate scars in most fossil archosaurs (e.g. non-avian theropods) examined in this study superficially resemble muscle scars in having a rugose texture indicative of soft tissue attachment. Clear and distinct muscle scars have been reported from fossil archosaurs ^1,2^. In the extant and fossil archosaur specimens we surveyed, we were unable to find any definite muscle scars on the posterior margin of the dorsal ribs that were comparable to those reported in the literature. In extant archosaurs, four muscles attach to the dorsal vertebral ribs in the vicinity of the uncinate process: m. iliocostalis, m. obliquus abdominis externus, mm. intercostales externi, mm. appendicocostales, and mm. intercostales interni ^3-6^. With the sole exception of m. obliquus abdominis externus, these muscles form wide sheets with fleshy or tendinous attachments to the dorsal vertebral ribs ^3,4,7,8^. Such extensive attachments would not be expected to leave well-defined, relatively small scars like the uncinate scars described in this study. Some of the muscle fibres of m. obliquus abdominis externus do converge on a small area, which might leave a well-defined scar in large-bodied archosaurs (e.g. non-avian dinosaurs). However, m. obliquus abdominus externus attaches to the lateral aspect of the dorsal vertebral ribs and the distal ends of the posterior most cervical vertebral ribs ^3-5^, while the features we identify as uncinate scars are consistently positioned on the posterior aspect of the dorsal ribs. Therefore, the features identified here as uncinate scars are unlikely to represent muscle scars because of their restricted extent, well-defined margins, and posterior anatomical position.

The slender strips that we identify as uncinate scars in ornithischians bear limited resemblance to the groove-like bite marks that have been found on bones of various fossil archosaurs ^9^. However, the slender strips reported in this study do not match the morphology or the distribution that might be expected for bite marks. Morphologically, the slender strips remain uniform in width along their full proximodistal extent, while the groove-like bite marks inflicted by carnivorous vertebrates such as non-avian theropods tend to be wide at the point of initial tooth-bone contact and taper towards the opposite end ^10,11^. As for distribution, the slender strips are consistently parallel to the long axes of the dorsal ribs and never number more than two, whereas bite marks would likely be far less regular in both orientation and number. As a result, we reject the interpretation that the slender strips found in this study might represent bite marks, alongside the interpretation that they might represent sites of muscle attachment and identify them decisively as uncinate scars comparable to those seen in extant archosaurs.

**Detailed description of the procedures used for the ancestral state reconstruction**

The distribution of uncinate processes was encoded in terms of a discrete character with three states: absence of uncinate processes (0), presence of cartilaginous uncinate processes (1), and presence of ossified uncinate processes (2). A total of five extant taxa and 36 fossil taxa were coded based on direct observation, and 17 taxa were coded based on information provided in the literature (Table 2). In one case, that of *Mariliasuchus amarali* from the Upper Cretaceous of Brazil ^12^, structures described in the literature as uncinate processes were not accepted as uncinate processes for the purposes of this study. The putative uncinate processes described in *M. amarali* are positioned between the two rib heads of each rib whereas the uncinate processes of other archosaurs are on the rib shafts. The putative uncinate processes do, however, resemble in both morphology and position the anterolateral process situated between the capitulum and tuberculum on the last cervical rib in extant crocodylians, and most certainly represent the anterolateral processes rather than true uncinate processes. Because there is no other evidence for uncinate processes in *M. amarali*, the presence of uncinate processes was coded as uncertain (?) for this taxon in the ancestral state reconstruction. The proterochampsian *Chanaresuchus bonapartei* was selected as a representative outgroup to Archosauria, and was assumed to lack uncinate processes based on the absence of uncinate scars on a total of 13 well preserved vertebral ribs observed in two individuals (MCZ 4037, 4038).

Two coding methods were used. Our preferred coding method scored ossified uncinate processes as present (2) if bony uncinate processes were known for a particular taxon; cartilaginous uncinate processes as present (1) if either cartilaginous uncinate processes (calcified or otherwise) were known, or uncinate scars were present but no uncinate processes were known; and the presence of uncinate processes as uncertain (?) if evidence of uncinate processes or scars was lacking. The alternate coding method was identical, except that absence of evidence was treated as evidence of absence beyond a certain threshold: uncinate processes were scored as absent (0) if more than five dorsal ribs were available for examination and no uncinate processes or uncinate scars were evident. Under the alternate coding method, nine taxa were scored as lacking uncinate processes.

The ancestral state reconstruction was performed on an informal supertree compiled from relevant phylogenetic studies, with a tree from Nesbitt ^13^ as a starting point. To add a phylogenetic tree to the informal supertree, at least one common taxon present in both trees was used as a topological landmark, and the new phylogenetic tree was grafted onto the informal supertree at the phylogenetic position of the common taxon (Table 3). *Chanaresuchus* *bonapartei* was chosen as a representative outgroup to Archosauria because multiple specimens could be examined in this study. To ensure that all taxa in which evidence of uncinate processes could be identified were included, five taxa from the original phylogenetic studies were replaced in the informal supertree, as follows: 1) *Aetosaurus ferratus* was replaced by Aetosauria indet. (NMMNH P50048); 2) MBR2747 within Phytosauria was replaced by Phytosauridae indet. (YPM 6699); 3) *Allosaurus* was replaced by *Allosaurus fragilis* (AMNH 5753); 4) *Apatosaurus* was replaced by *Apatosaurus excelsus* (YPM 1980 and YPM 1981); and 5) *Crypturellus undulatus* was replaced by *Rhea americana* (UAMZ 5019). Temporal ranges for taxa included in the supertree were estimated based on first and last appearance data, primarily taken from the Paleobiology Database ^14^. For taxa without data in the Paleobiology Database, data were taken from the literature (Table 4). Branch lengths were then estimated using the temporal ranges and the Paleotree ^15^ package in RStudio 4.1.2. The ancestral state reconstruction was carried out both with and without taking estimated branch lengths into account, using the same informal supertree in both cases. To facilitate the calculations, polytomies were resolved into branches with lengths of one. Polytomies were resolved sequentially, according to the order in which taxa listed first in the tree file were accordingly resolved in relatively basal positions.

The ancestral state reconstruction was performed in RStudio 4.1.2 using the Phangorn package for maximum parsimony analysis ^16^, the APE package for maximum likelihood analysis ^17^, and the MBASR toolkit for Bayesian inference using MRBayes ^18^. The results of the ancestral state reconstructions were visualized and annotated with ggtree ^19^.

**Table 2. List of character codings used in the ancestral state reconstruction.**

| Taxa | Specimen or reference used as basis for codings | Preferred coding | Alternate coding |
| --- | --- | --- | --- |
| *Chanaresuchus* (based on *Chanaresuchus bonapartei*) | MCZ 4035, MCZ 4036, MCZ 4037, MCZ 4038 | 0 | 0 |
| *Caiman crocodilus* | ROM R6872, ROM R7707, UAMZ unnumbered | 1 | 1 |
| *Alligator mississippiensis* | ROM R4410 and ROM R4406 | 1 | 1 |
| *Alligator sinensis* | ^4^ | 1 | 1 |
| *Crocodylus siamensis* (farm crocodile) | unnumbered specimen dissected at Lingshui Crocodile Farm, Hainan Province, China | 1 | 1 |
| *Crocodylus acutus* | CMN 10018 | 1 | 1 |
| Phytosauria indet. | YPM 6699 | 1 | 1 |
| Aetosauria indet. | NMMNH P50048 | 1 | 1 |
| *Lotosaurus adentus* | IVPP V 4910 | ? | 0 |
| *Protosuchus richardsoni* | AMNH 3024 | ? | 0 |
| *Araripesuchus gomesii* | AMNH 24450 | 1 | 1 |
| *Penghusuchus pani* | ^20^ | 1 | 1 |
| Lagerpetidae (based on *Lagerpeton chanarensis*) | MCZ 4121 | ? | 0 |
| *Rhea americana* | UAMZ 5019 | 2 | 2 |
| *Gallus gallus* | ^6^ | 2 | 2 |
| *Chauna torquata* | ^21^ | 0 | 0 |
| *Lithornis* | ^22^ | 2 | 2 |
| *Deinonychus* *antirrhopus* | ^23^ | 2 | 2 |
| *Velociraptor* *mongoliensis* | ^24^ | 2 | 2 |
| *Saurornitholestes langstoni* | TMP1988.121.0039 and UALVP 55700 | 2 | 2 |
| *Microraptor* *zhaoianus* | ^25^ | 2 | 2 |
| *Caudipteryx* *zoui* | ^26^ | 2 | 2 |
| *Oviraptor philoceratops* | ^27^ | 2 | 2 |
| *Conchoraptor gracilis* | ^28^ | 2 | 2 |
| *Struthiomimus altus* | AMNH 5355 | 1 | 1 |
| *Pelecanimimus polyodon* | ^29^ | 2 | 2 |
| *Gorgosaurus libratus* | UALVP 10 | 1 | 1 |
| *Daspletosaurus torosus* | CMN 8506 | 1 | 1 |
| *Albertosaurus sarcophagus* | TMP 99.50.41 and TMP 99.50.43 | 1 | 1 |
| *Allosaurus fragilis* | AMNH 5753 | 1 | 1 |
| *Apatosaurus excelsus* | YPM 1980 and YPM 1981 | 1 | 1 |
| *Camarasaurus* (based on  *Camarasaurus grandis*) | YPM1905 | ? | 0 |
| *Plateosaurus quenstedti* | MCZ 2483 | ? | 0 |
| *Anchisaurus* (based on  *Anchisaurus colurus*) | YPM 1883 | ? | 0 |
| *Parasaurolophus walkeri* | ROM 1215 | ? | 0 |
| *Edmontosaurus regalis* | CMN 2289 | ? | 0 |
| *Gryposaurus latidens* | AMNH 5465 | 1 | 1 |
| *Gryposaurus notabilis* | AMNH 5350 | 1 | 1 |
| *Bactrosaurus johnsoni* | AMNH 6553 | 1 | 1 |
| *Camptosaurus dispar* | YPM 1877 and YPM 1880 | ? | 0 |
| *Tenontosaurus tilleti* | AMNH 3040 | 1 | 1 |
| *Zephyrosaurus schaffi* | MCZ 4392 | 1 | 1 |
| *Parksosaurus warreni* | ROM 804 | 1 | 1 |
| *Thescelosaurus* *assiniboiensis* | ^30^ | 1 | 1 |
| *Talenkauen santacrucensis* | ^31^ | 1 | 1 |
| *Hypsilophodon foxii* | ^32^ | 1 | 1 |
| *Pachyrhinosaurus* *lakustai* | UALVP 57289 | 1 | 1 |
| *Centrosaurus* sp. | TMP 82.18.16, TMP 82.19.41, TMP 96.176.135, TMP 82.18.56, and TMP82.18.281 | 1 | 1 |
| *Chasmosaurus belli* | ROM 843 | ? | 0 |
| *Protoceratops andrewsi* | AMNH 6416 | ? | 0 |
| *Leptoceratops* *gracilis* | CMN 8889 | 1 | 1 |
| *Euoplocephalus tutus* | AMNH 5337 | 1 | 1 |
| *Saichania chulsanensis* | ^33^ | 1 | 1 |
| *Panoplosaurus mirus* | ROM 1215 | 1 | 1 |
| *Sauropelta edwardsorum* | AMNH 3032 | 1 | 1 |
| *Stegosaurus stenops* | AMNH 650, AMNH 5752, and YPM 1856 | 1 | 1 |
| *Huayangosaurus* *taibaii* | ^34^ | 1 | 1 |

**Table 3. List of phylogenetic studies used in compiling the informal supertree**

| Expansion of informal supertree | Key taxa present in two references | Reference |
| --- | --- | --- |
| Basal avemetatarsalians only | NA | Strict consensus, Nesbitt dataset ^35^ |
| 1) Basal avemetatarsalians to silesaurids  2) Basal avemetatarsalians to basal sauropodomorphs | *Marasuchus lilloensis* | Strict consensus, Nesbitt dataset ^35^  Strict consensus, fifth analysis* ^36^ |
| 1) Basal sauropodomorphs to plateosaurids  2) Basal sauropodomorphs to basal massopodans | *Pantydraco caducus*  *Thecodontosaurus antiquus* | Strict consensus, fifth analysis ^36^  Strict consensus, ^37^ |
| Basal massopodans to basal eusauropods | *Shunosaurus* | Strict consensus ^37^  Strict consensus, with implied weight ^38^ |
| Basal somphospondylians to titanosaurs | *Chubutisaurus insignis*  *Andesaurus delgadoi* | Strict consensus, with implied weight ^38^  Majority rule ^39^ |
| Basal saurischians to basal theropods | *Eoraptor lunensis* | Strict consensus, fifth analysis ^36^  Strict consensus ^40^ |
| Basal theropods to basal coelurosaurs | *Allosaurus* | Strict consensus ^40^  Strict consensus ^41^ |
| Basal coelurosaurs to ornithomimosaurs | *Nqwebasaurus thwazi* | Strict consensus ^41^  Strict consensus ^29^ |
| Basal maniraptorans to pennaraptorans** | *Yi qi*  *Epidexipteryx hui* | Strict consensus ^41^  Reduced strict consensus ^42^ |
| 1) Basal dinosauriforms to basal ornithischians  2) Basal dinosauriforms to heterodontosaurids | *Eoraptor lunensis* | Strict consensus, fifth analysis ^36^  Strict consensus ^43^ |
| Basal ornithischians to stegosaurs | *Scutellosaurus lawleri* | Strict consensus ^43^  Strict consensus ^44^ |
| Basal ornithischians to basal ankylosaurs | *Gargoyleosaurus parkpinorum* | Strict consensus ^43^  Majority rule ^45^ |
| Basal ankylosaurs to ankylosaurids | Nodosauridae | Majority rule ^45^  Strict consensus ^46^ |
| Basal ornithischians to basal neornithischians | *Lesothosaurus diagnosticus*  *Agilisaurus louderbacki* | Strict consensus ^43^  Strict consensus ^47^ |
| Basal ornithopods to basal iguanodontians | *Hypsilophodon foxii*  *Rhabdodon* sp. | Strict consensus ^47^  Majority rule ^48^ |
| Basal hadrosauromorphs to hadrosaurids | *Hadrosaurus foulkii* | Majority rule ^48^  Strict consensus ^49^ |
| Basal neornithischians to ceratopsids | *Yinlong downsi* | Strict consensus ^43^  Strict consensus ^50^ |
| Basal neornithischians to pachycephalosaurs | *Wannanosaurus yansiensis* | Strict consensus ^43^  Strict consensus ^51^ |
| Basal archosaurs to phytosaurs*** | *Parasuchus hislopi* | Strict consensus, Nesbitt dataset ^35^  Strict consensus ^52^ |
| Basal archosaurs to basal pseudosuchians (excluding phytosaurs) | NA | Reduced strict consensus ^53^ |
| Basal pseudosuchians to basal crocodylomorphs | *Postosuchus* | Reduced strict consensus ^53^  Strict consensus ^54^ |
| Basal crocodylomorphs to basal crocodyliforms | *Dibothrosuchus elaphros* | Strict consensus ^54^  Strict consensus ^55^ |
| 1) Basal crocodyliforms to notosuchians  2) Basal crocodyliforms to eusuchians | *Hsisosuchus* | Strict consensus ^55^  Strict consensus, first analysis ^56^ |
| Basal notosuchians to sebecosuchians | *Chimaerasuchus paradoxus* | Strict consensus, first analysis ^56^  Reduced consensus ^57^ |
| Basal neosuchians to gonipholidids | *Sunosuchus junggarensis* | Strict consensus, first analysis ^56^  Strict consensus, first analysis ^58^ |
| Basal eusuchians to crocodylians | *Bernissartia fagesii* | Strict consensus, first analysis ^56^  Strict consensus, analysis 1.3 ^59^ |

*The fifth analysis was chosen because it includes more taxa and internal nodes, and recovering cartilaginous uncinate processes at node Avemetatarsalia is more difficult when many taxa rather than few taxa are present in the basal avemetatarsalian part of the tree (i.e. there are more steps for the ancestral state reconstruction to evaluate, which increases the likelihood of recovering uncinate processes as absent or unknown when all other factors remain the same). ** Placement of Pennaraptora within Maniraptora follows Pittman, et al. ^42^. *** We followed Nesbitt, et al. ^35^ in positioning Phytosauria as the basalmost clade within Pseudosuchia, although recent studies have recovered phytosaurs outside Archosauria ^60,61^. See discussion in the main text for the implications of the phylogenetic position of Phytosauria for the reconstructed origin of uncinate processes.

**Table 4. First and last appearance data for taxa used in the ancestral state reconstruction.**

| Taxon* | First appearance time (Ma) | Last appearance time (Ma) |
| --- | --- | --- |
| El_Chocón_rebbachisaurid ^62^ | 129.4 | 100.5 |
| IOW_rebbachisaurid_caudal ^63^ | 129.4 | 125 |
| CV00214 ^64^ | 163.5 | 152.1 |
| EK_troodontid_IGM_10044** | 167.7 | 66 |
| *Archaeopteryx*_Eichstätt *** | 152.1 | 125.45 |
| *Archaeopteryx*_Thermopolis *** | 152.1 | 125.45 |
| *Archaeopteryx*_Berlin *** | 152.1 | 125.45 |
| *Archaeopteryx*_London *** | 152.1 | 125.45 |
| *Archaeopteryx*_11^th^ *** | 152.1 | 125.45 |
| *Archaeopteryx*_Munich *** | 152.1 | 125.45 |
| *Archaeopteryx*_Solnhofen *** | 152.1 | 125.45 |
| *Minmi*_sp. ^45^ | 145 | 100.5 |
| *Antarctopelta* ^65^ | 84.9 | 70.6 |
| Argentinian_nodosaurid ** | 157.3 | 66 |
| *Zhejiangosaurus* ^66^ | 99.7 | 94.3 |
| Kaiparowits_orodromine ^67^ | 76.6 | 74.3 |
| *Camptosaurus*_*valdensis* ^68^ | 136.4 | 122.46 |
| *Orthomerus_dolloi* ^69^ | 70.6 | 66 |
| *Mercuriceratops_gemini* ^70^ | 84.9 | 70.6 |
| *Mojoceratops_kaiseni* ^71^ | 85.8 | 70.6 |
| *Nedoceratops_hatcheri* ^72^ | 69 | 66 |
| *Foraminacephale_brevis* ^73^ | 85.8 | 70.6 |
| *Sinocephale_bexelli* ^74^ | 92 | 66 |
| *Paleorhinus_parvus* ^75^ | 235 | 221.5 |
| NMMNHS_P4781 ^76^ | 208.5 | 201.3 |
| TMM_31100_1332 ^77^ | 228 | 220 |
| USNM_v_21376 ^78^ | 237 | 208.5 |
| PEFO_34852 ^79^ | 237 | 201.3 |
| *Machaeroprosopus_zunii* ^77^ | 237 | 208.5 |
| USNM_v_17098 ^80^ | 237 | 227 |
| NMMNHS_P4256 ^81^ | 227 | 208.5 |
| NMMNHS_P31094 ^82^ | 221 | 206 |
| Phytosauria_indet. ** | 237 | 196.5 |
| *Mystriosuchus_steinbergeri* ^52^ | 216 | 211 |
| *Ornithosuchus_longidens* ^83^ | 235 | 205 |
| Waldshut_taxon ^84^ | 247.2 | 242 |
| CM_73372 ^85^ | 227 | 208.5 |
| UCMP_97638 ^86^ | 196.5 | 183 |
| *Notochampsa_istedana* ^87^ | 182.28 | 180.1 |
| Lumbrera_form ^88^ | 56 | 41.2 |
| *Hyposaurus_rogersii* ^89^ | 70.6 | 61.7 |
| PIN_4174_1 ^90^ | 152.1 | 145 |
| *Kansajasuchus_extensus* ^91^ | 100.5 | 66 |
| *Hulkeopholis_plotos* ^92^ | 117 | 103 |
| *Hulkeopholis_willetti* ^93^ | 140.2 | 136.4 |
| Dollo_s_*Anteophthalmosuchus* ^58^ | 129.4 | 122.46 |
| *Paralligator_tersus* ^94^ | 72.1 | 66 |
| *Paralligator_ancestralis* ^95^ | 75 | 80 |
| UCMP_39978 ^96^ | 38 | 7.246 |
| *Tomistoma_cairense* ^97^ | 47.8 | 41.2 |
| *Crocodylus_affinis* ^98^ | 50.3 | 47.8 |
| *Osteolaemus_tetraspis* **** | 0 | 0 |
| *Mecistops_cataphractus***** | 0 | 0 |
| *Crocodylus_johnstoni* **** # | 0 | 0 |
| *Crocodylus_novaeguineae* **** | 0 | 0 |
| *Crocodylus_mindorensis* **** | 0 | 0 |
| *Crocodylus_moreletii* **** | 0 | 0 |
| *Crocodylus_rhombifer* **** | 0 | 0 |
| *Crocodylus_acutus* **** | 0 | 0 |
| *Crocodylus_intermedius* **** | 0 | 0 |
| *Crocodylus_porosus* **** | 0 | 0 |
| *Crocodylus_siamensis* **** | 0 | 0 |
| *Crocodylus_niloticus* **** | 0 | 0 |
| *Crocodylus_palustris* **** | 0 | 0 |
| *Alligator_sinensis* **** | 0 | 0 |
| *Alligator_mississippiensis* **** | 0 | 0 |
| *Caiman_lutescens* **** | 0 | 0 |
| *Caiman_yacare* **** | 0 | 0 |
| *Gavialis_gangeticus* **** | 0 | 0 |
| *Melanosuchus_niger* **** | 0 | 0 |
| *Caiman_latirostris* **** | 0 | 0 |
| *Caiman_crocodilus* **** | 0 | 0 |
| *Paleosuchus_palpebrosus* **** | 0 | 0 |
| *Paleosuchus_trigonatus* **** | 0 | 0 |
| *Tomistoma_schlegelii* **** | 0 | 0 |
| *Rhea_americana* **** | 0 | 0 |
| *Chauna_torquata* **** | 0 | 0 |
| *Anas_platyrhynchos* **** | 0 | 0 |
| *Pauxi_pauxi* **** # | 0 | 0 |
| *Gallus_gallus* **** | 0 | 0 |

*For most taxa, first and last appearance data used to estimate branch length were obtained from the Paleobiology Database. ** First and last appearance data of Troodontidae, Nodosauridae, and Mystriosuchinae were used for IGM_10044, Argentinian_nodosaurid, and Phytosauria_indet, respectively. *** same ages used for all *Archaeopteryx*. **** branch lengths of extant taxa were assigned values of zero. # indicates misspelled taxonomic names in the raw results in the Supplementary data. Spelling in this document is most up to date.

**Table 5. Probabilities of the three ancestral states in selected archosaur clades**

| Clade  ASR  methods | Archosauria  (Node 1027) | Dinosauria  (Node 1047) | Saurischia  (Node 1048) | Theropoda (Node 1205) | Maniraptoriformes  (Node 1286) | Pennaraptora  (Node 1318) |
| --- | --- | --- | --- | --- | --- | --- |
| MP with preferred coding | S0: 0  S1: 1  S2: 0 | S0: 0  S1: 1  S2: 0 | S0: 0  S1: 1  S2: 0 | S0: 0  S1: 1  S2: 0 | S0: 0  S1: 0  S2: 1 | S0: 0  S1: 0  S2: 1 |
| MP with preferred coding and branch lengths | S0: 0  S1: 1  S2: 0 | S0: 0  S1: 1  S2: 0 | S0: 0  S1: 1  S2: 0 | S0: 0  S1: 1  S2: 0 | S0: 0  S1: 0  S2: 1 | S0: 0  S1: 0  S2: 1 |
| ML with preferred coding | S0: 0.02  S1: 0.98  S2: 0 | S0: 0  S1: 1  S2: 0 | S0: 0  S1: 1  S2: 0 | S0: 0  S1: 1  S2: 0 | S0: 0  S1: 0.10  S2: 0.90 | S0: 0  S1: 0  S2: 1 |
| ML with preferred coding and branch lengths | S0: 0  S1: 1  S2: 0 | S0: 0  S1: 1  S2: 0 | S0: 0  S1: 1  S2: 0 | S0: 0  S1: 1  S2: 0 | S0: 0  S1: 0.02  S2: 0.98 | S0: 0  S1: 0  S2: 1 |
| MB with preferred coding | S0: 0.33  S1: 0.33  S2: 0.33 | S0: 0.33  S1: 0.33  S2: 0.33 | S0: 0.33  S1: 0.33  S2: 0.33 | S0: 0.33  S1: 0.33  S2: 0.33 | S0: 0.33  S1: 0.33  S2: 0.33 | S0: 0.33  S1: 0.33  S2: 0.33 |
| MB with preferred coding and branch lengths | S0: 0.23  S1: 0.53  S2: 0.23 | S0: 0.33  S1: 0.34  S2: 0.33 | S0: 0.33  S1: 0.34  S2: 0.33 | S0: 0.33  S1: 0.33  S2: 0.33 | S0: 0.33  S1: 0.33  S2: 0.33 | S0: 0.33  S1: 0.33  S2: 0.33 |
| MP with alternate coding | S0: 0  S1: 1  S2: 0 | S0: 0  S1: 1  S2: 0 | S0: 0  S1: 1  S2: 0 | S0: 0  S1: 1  S2: 0 | S0: 0  S1: 0  S2: 1 | S0: 0  S1: 0  S2: 1 |
| MP with alternate coding and branch lengths | S0: 0  S1: 1  S2: 0 | S0: 0  S1: 1  S2: 0 | S0: 0  S1: 1  S2: 0 | S0: 0  S1: 1  S2: 0 | S0: 0  S1: 0  S2: 1 | S0: 0  S1: 0  S2: 1 |
| ML with alternate coding | S0: 0.92  S1: 0.08  S2: 0 | S0: 0.41  S1: 0.57  S2: 0.01 | S0: 0.41  S1: 0.57  S2: 0.02 | S0: 0.35  S1: 0.61  S2: 0.04 | S0: 0.01  S1: 0.09  S2: 0.90 | S0: 0  S1: 0.01  S2: 0.99 |
| ML with alternate coding and branch lengths | S0: 0.26  S1: 0.61  S2: 0.13 | S0: 0.34  S1: 0.37  S2: 0.29 | S0: 0.34  S1: 0.36  S2: 0.3 | S0: 0.34  S1: 0.34  S2: 0.32 | S0: 0.33  S1: 0.33  S2: 0.33 | S0: 0.33  S1: 0.33  S2: 0.33 |
| MB with alternate coding | S0: 0.33  S1: 0.33  S2: 0.33 | S0: 0.33  S1: 0.33  S2: 0.33 | S0: 0.33  S1: 0.33  S2: 0.33 | S0: 0.33  S1: 0.33  S2: 0.33 | S0: 0.33  S1: 0.33  S2: 0.33 | S0: 0.33  S1: 0.33  S2: 0.33 |
| MB with alternate coding and branch lengths | S0: 0.24  S1: 0.55  S2: 0.21 | S0: 0.33  S1: 0.34  S2: 0.33 | S0: 0.33  S1: 0.34  S2: 0.33 | S0: 0.33  S1: 0.33  S2: 0.33 | S0: 0.33  S1: 0.33  S2: 0.33 | S0: 0.33  S1: 0.33  S2: 0.33 |

Abbreviations: ASR, ancestral state reconstruction; MB, Bayesian inference using Mr. Bayes; ML, Maximum likelihood; MP, Maximum parsimony; S, state.

**Supplementary References**

1 Carrano, M. T. & Hutchinson, J. R. Pelvic and hindlimb musculature of *Tyrannosaurus rex* (Dinosauria: Theropoda). *J. Morphol.* **253**, 207-228; 10.1002/jmor.10018 (2002).

2 Dumbravă, M., Codrea, V., Solomon, A. & Andrei, R. Hind leg myology of the Maastrichtian (Latest Cretaceous) euornithischian dinosaur *Zalmoxes shquiperorum* from the Hațeg Basin, Romania: Preliminary data. *Olten.-Stud. comun., Ştiinţ. nat.* **29**, 7-18 (2013).

3 Shufeldt, R. W. The musculature of the trunk in *The myology of the raven (Corvus corax sinuatus): A guide to the study of the muscular system in birds* 258-318 (Macmillan and Company, 1890)

4 Cong, L. Y., Hou, L. H. & Wu, X. C. *The gross anatomy of Alligator sinensis Fauvel: Integument, osteology, and myology (in Chinese with English summary)* 1-388 (China Science Publishing & Media Ltd., 1988)

5 Frey, T. V. E. Anatomie des Körperstammes von *Alligator mississippiensis* Daudin. *Stuttg. Beitr. Nat. A* **424**, 1-106 (1988).

6 Ghetie, V. *Atlas de anatomie a păsărilor domestice* 1-295 (Academiei Republicii Socialiste România, 1976)

7 Codd, J. R. *et al.* A novel accessory respiratory muscle in the American alligator (*Alligator mississippiensis*). *Biol. Lett.* **15**, 20190354; 10.1098/rsbl.2019.0354 (2019).

8 Rose, K. A. R. *et al.* Scaling of axial muscle architecture in juvenile *Alligator mississippiensis* reveals an enhanced performance capacity of accessory breathing mechanisms. *J. Anat.* **6**, 1 - 14; 10.1111/joa.13523 (2021).

9 Hone, D. W. E. & Rauhut, O. W. M. Feeding behaviour and bone utilization by theropod dinosaurs. *Lethaia* **43**, 232-244 (2010).

10 Tanke, D. H. & Currie, P. J. Head-biting behavior in theropod dinosaurs: Paleopathological evidence. *Gaia* **15**, 167-184 (1998).

11 Rivera-Sylva, H. E., Hone, D. W. E. & Dodson, P. Bite marks of a large theropod on an hadrosaur limb bone from Coahuila, Mexico. *Boletin de la Soc. Geol. Mex.* **64**, 155-159 (2012).

12 Nobre, P. H. & Carvalho, I. d. S. Postcranial skeleton of *Mariliasuchus amarali* Carvalho and Bertini, 1999 (Mesoeucrocodylia) from the Bauru Basin, Upper Cretaceous of Brazil. *Ameghiniana* **50**, 98-113; 10.5710/amgh.15.8.2012.500 (2013).

13 Nesbitt, S. J. The early evolution of archosaurs: relationships and the origin of major clades. *Bull. Am. Mus. Nat. Hist.* **352**, 1-292 (2011).

14 Peters, S. E. & McClennen, M. The Paleobiology Database application programming interface. *Paleobiology* **42**, 1-7; 10.1017/pab.2015.39 (2015).

15 Bapst, D. W. Paleotree: An r package for paleontological and phylogenetic analyses of evolution. *Methods Ecol. Evol.* **3**, 803-807; 10.1111/j.2041-210X.2012.00223.x (2012).

16 Schliep, K. P. Phangorn: Phylogenetic analysis in R. *Bioinformatics* **27**, 592-593; 10.1093/bioinformatics/btq706 (2011).

17 Paradis, E., Claude, J. & Strimmer, K. APE: Analyses of Phylogenetics and Evolution in R language. *Bioinformatics* **20**, 289-290; 10.1093/bioinformatics/btg412 (2004).

18 Heritage, S. MBASR: Workflow-simplified ancestral state reconstruction of discrete traits with MrBayes in the R environment. *bioRxiv*; 10.1101/2021.01.10.426107 (2021).

19 Yu, G. *et al.* ggtree : An r package for visualization and annotation of phylogenetic trees with their covariates and other associated data. *Methods Ecol. Evol.* **8**, 28-36; 10.1111/2041-210x.12628 (2016).

20 Shan, H. Y., Wu, X. C., Cheng, Y. N. & Sato, T. A new tomistomine (Crocodylia) from the Miocene of Taiwan. *Can. J. Earth Sci.* **46**, 529-555; 10.1139/e09-036 (2009).

21 Carboneras, C. Order Anseriformes in *Handbook of birds of the world* (ed. Hoyo, J. d. *et al.*) 528-628 (Lynx Edicions, 1992)

22 Bourdon, E. & Lindow, B. A redescription of *Lithornis vulturinus* (aves, palaeognathae) from the Early Eocene Fur Formation of Denmark. *Zootaxa* **4032**, 493-514; 10.11646/zootaxa.4032.5.2 (2015).

23 Ostrom, J. H. Osteology of *Deinonychus antirrhopus*, an unusual theropod from the Lower Cretaceous of Montana. *Bull. Peabody Mus. Nat. Hist.* **30**, 1-165 (1969).

24 Norell, M. A. & Makovicky, P. J. Important features of the dromaeosaurid skeleton II: Information from newly collected specimens of *Velociraptor mongoliensis*. *Am. Mus. Novit.* **3282**, 1-48 (1999).

25 Xu, X., Zhou, Z. H. & Wang, X. L. The smallest known non-avian theropod dinosaur. *Nature* **408**, 705-708 (2000).

26 Zhou, Z. H., Wang, X. L., Zhang F. C. & Xu, X. Important features of *Caudipteryx* - evidence from two nearly complete new specimens. *Vert. PalAs.* **38**, 241-254 (2000).

27 Codd, J. R., Manning, P. L., Norell, M. A. & Perry, S. F. Avian-like breathing mechanics in maniraptoran dinosaurs. *Proc. R. Soc. Lond., Ser. B: Biol. Sci.* **275**, 157-161; 10.1098/rspb.2007.1233 (2008).

28 Barsbold, R., Maryańska, T. & Osmólska, H. Oviraptorosauria in *The Dinosauria* (ed. Weishampel, D. B. *et al.*) 249-258 (University of California Press, 2007)

29 Cuesta, E., Vidal, D., Ortega, F., Shibata, M. & Sanz, J. L. *Pelecanimimus* (Theropoda: Ornithomimosauria) postcranial anatomy and the evolution of the specialized manus in ornithomimosaurs and sternum in maniraptoriforms. *Zool. J. Linn. Soc.*, 1-39; 10.1093/zoolinnean/zlab013/6271061 (2021).

30 Brown, C. M., Boyd, C. A. & Russell, A. P. A new basal ornithopod dinosaur (Frenchman Formation, Saskatchewan, Canada), and implications for Late Maastrichtian ornithischian diversity in North America. *Zool. J. Linn. Soc.* **163**, 1157-1198; 10.1111/j.1096-3642.2011.00735.x (2011).

31 Novas, F. E., Cambiaso, A. V. & Ambrosio, A. A new basal iguanodontian (Dinosauria, Ornithischia) from the Upper Cretaceous of Patagonia. *Ameghiniana* **41**, 75-82 (2004).

32 Butler, R. J. & Galton, P. M. The ‘dermal armour’ of the ornithopod dinosaur *Hypsilophodon* from the Wealden (Early Cretaceous: Barremian) of the Isle of Wight: A reappraisal. *Cretac. Res.* **29**, 636-642; 10.1016/j.cretres.2008.02.002 (2008).

33 Maryañska, T. Ankylosauridae (Dinosauria) from Mongolia. *Acta Palaeontol. Pol.* **37**, 85-151 (1977).

34 Zhou, S. W. A nearly complete skeleton of a stegosaur from the Middle Jurassic of Dashanpu, Zigong, Sichuan. *J. Chengdu Univ. Technol.* **1**, 15-26 (1983).

35 Nesbitt, S. J. *et al.* The earliest bird-line archosaurs and the assembly of the dinosaur body plan. *Nature* **544**, 484-487; 10.1038/nature22037 (2017).

36 Müller, R. T. *et al.* Early evolution of sauropodomorphs: Anatomy and phylogenetic relationships of a remarkably well-preserved dinosaur from the Upper Triassic of southern Brazil. *Zool. J. Linn. Soc.* **184**, 1187-1248 (2018).

37 Otero, A. & Pol, D. Postcranial anatomy and phylogenetic relationships of *Mussaurus patagonicus* (Dinosauria, Sauropodomorpha). *J. Vertebr. Paleontol.* **33**, 1138-1168; 10.1080/02724634.2013.769444 (2013).

38 Mannion, P. D., Upchurch, P., Schwarz, D. & Wings, O. Taxonomic affinities of the putative titanosaurs from the late Jurassic Tendaguru Formation of Tanzania: Phylogenetic and biogeographic implications for eusauropod dinosaur evolution. *Zool. J. Linn. Soc.* **185**, 784-909 (2019).

39 Gorscak, E. & O'Connor, P. M. A new African titanosaurian sauropod dinosaur from the middle Cretaceous Galula Formation (Mtuka Member), Rukwa Rift Basin, southwestern Tanzania. *PLOS One* **14**, e0211412; 10.1371/journal.pone.0211412 (2019).

40 Carrano, M. T., Benson, R. B. J. & Sampson, S. D. The phylogeny of Tetanurae (Dinosauria: Theropoda). *J. Syst. Palaeontol.* **10**, 211-300; 10.1080/14772019.2011.630927 (2012).

41 Pol, D. & Goloboff, P. A. The impact of unstable taxa in coelurosaurian phylogeny and resampling support measures for parsimony analyses. *Bull. Am. Mus. Nat. Hist.* **440**, 97-115 (2020).

42 Pittman, M. *et al.* Pennaraptoran systematics. *Bull. Am. Mus. Nat. Hist.* **440**, 7-36 (2020).

43 Han, F., Forster, C. A., Xu, X. & Clark, J. M. Postcranial anatomy of *Yinlong downsi* (Dinosauria: Ceratopsia) from the Upper Jurassic Shishugou Formation of China and the phylogeny of basal ornithischians. *J. Syst. Palaeontol.* **16**, 1159-1187; 10.1080/14772019.2017.1369185 (2018).

44 Raven, T. J. & Maidment, S. C. R. A new phylogeny of Stegosauria (Dinosauria, Ornithischia). *Palaeontology* **60**, 401-408 (2017).

45 Arbour, V. M. & Currie, P. J. Systematics, phylogeny and palaeobiogeography of the ankylosaurid dinosaurs. *J. Syst. Palaeontol.* **14**, 385-444; 10.1080/14772019.2015.1059985 (2016).

46 Arbour, V. M. & Evans, D. C. A new ankylosaurine dinosaur from the Judith River Formation of Montana, USA, based on an exceptional skeleton with soft tissue preservation. *R. Soc. Open Sci.* **4**, 161086; 10.1098/rsos.161086 (2017).

47 Madzia, D., Boyd, C. A. & Mazuch, M. A basal ornithopod dinosaur from the Cenomanian of the Czech Republic. *J. Syst. Palaeontol.* **16**, 967-979; 10.1080/14772019.2017.1371258 (2018).

48 Madzia, D., Jagt, J. W. M. & Mulder, E. W. A. Osteology, phylogenetic affinities and taxonomic status of the enigmatic Late Maastrichtian ornithopod taxon *Orthomerus dolloi* (Dinosauria, Ornithischia). *Cretac. Res.* **108**; 10.1016/j.cretres.2019.104334 (2020).

49 McDonald, A. T., Wolfe, D. G., Freedman Fowler, E. A. & Gates, T. A. A new brachylophosaurin (Dinosauria: Hadrosauridae) from the Upper Cretaceous Menefee Formation of New Mexico. *PeerJ* **9**, e11084; 10.7717/peerj.11084 (2021).

50 Yu, C., Prieto-Marquez, A., Chinzorig, T., Badamkhatan, Z. & Norell, M. A neoceratopsian dinosaur from the Early Cretaceous of Mongolia and the early evolution of Ceratopsia. *Commun. Biol.* **3**, 499; 10.1038/s42003-020-01222-7 (2020).

51 Evans, D. C., Brown, C. M., You, H. L. & Campione, N. E. Description and revised diagnosis of Asia’s first recorded pachycephalosaurid, *Sinocephale bexelli* gen. nov., from the Upper Cretaceous of Inner Mongolia, China. *Can. J. Earth Sci.* **58**, 981-992; 10.1139/cjes-2020-0190 (2021).

52 Butler, R. J. *et al.* Description and phylogenetic placement of a new marine species of phytosaur (Archosauriformes: Phytosauria) from the Late Triassic of Austria. *Zool. J. Linn. Soc.* **187**, 198-228 (2019).

53 Roberto-Da-Silva, L., Müller, R. T., França, M. A. G. d., Cabreira, S. F. & Dias-Da-Silva, S. An impressive skeleton of the giant top predator *Prestosuchus chiniquensis* (Pseudosuchia: Loricata) from the Triassic of southern Brazil, with phylogenetic remarks. *Hist. Biol.*, 1-20; 10.1080/08912963.2018.1559841 (2018).

54 Leardi, J. M., Pol, D. & Clark, J. M. Detailed anatomy of the braincase of *Macelognathus vagans* Marsh, 1884 (Archosauria, Crocodylomorpha) using high resolution tomography and new insights on basal crocodylomorph phylogeny. *PeerJ* **5**, e2801; 10.7717/peerj.2801 (2017).

55 Dollman, K. N., Viglietti, P. A. & Choiniere, J. N. A new specimen of *Orthosuchus stormbergi* (Nash 1968) and a review of the distribution of southern African Lower Jurassic crocodylomorphs. *Hist. Biol.* **31**, 653-664; 10.1080/08912963.2017.1387110 (2017).

56 Rummy, P. *et al.* A new paralligatorid (Crocodyliformes, Neosuchia) from the mid-Cretaceous of Jilin Province, northeastern China. *Cretac. Res.* **129**; 10.1016/j.cretres.2021.105018 (2022).

57 Selles, A. G. *et al.* A small Cretaceous crocodyliform in a dinosaur nesting ground and the origin of sebecids. *Sci. Rep.* **10**, 15293; 10.1038/s41598-020-71975-y (2020).

58 Ristevski, J., Young, M. T., de Andrade, M. B. & Hastings, A. K. A new species of *Anteophthalmosuchus* (Crocodylomorpha, Goniopholididae) from the Lower Cretaceous of the Isle of Wight, United Kingdom, and a review of the genus. *Cretac. Res.* **84**, 340-383; 10.1016/j.cretres.2017.11.008 (2018).

59 Rio, J. P. & Mannion, P. D. Phylogenetic analysis of a new morphological dataset elucidates the evolutionary history of Crocodylia and resolves the long-standing gharial problem. *PeerJ* **9**, e12094; 10.7717/peerj.12094 (2021).

60 Ezcurra, M. D. The phylogenetic relationships of basal archosauromorphs, with an emphasis on the systematics of proterosuchian archosauriforms. *PeerJ* **4**, e1778; 10.7717/peerj.1778 (2016).

61 Marsh, A. D., Smith, M. E., Parker, W. G., Irmis, R. B. & Kligman, B. T. Skeletal anatomy of *Acaenasuchus geoffreyi* Long and Murry, 1995 (Archosauria: Pseudosuchia) and its implications for the origin of the aetosaurian carapace. *J. Vertebr. Paleontol.* **40**; 10.1080/02724634.2020.1794885 (2020).

62 Carabajal, A. P., Canale, J. I. & Haluza, A. New rebbachisaurid cranial remains (Sauropoda, Diplodocoidea) from the Cretaceous of Patagonia, Argentina, and the first endocranial description for a South American representative of the clade. *J. Vertebr. Paleontol.* **36**; 10.1080/02724634.2016.1167067 (2016).

63 Mannion, P. D. A rebbachisaurid sauropod from the Lower Cretaceous of the Isle of Wight, England. *Cretac. Res.* **30**, 521-526; 10.1016/j.cretres.2008.09.005 (2009).

64 Dong, Z. M., Zhou, S. W. & Zhang, Y. H. Dinosaurs from the Jurassic of Sichuan *Palaeontol. Sin.* **162**, 1-151 (1983).

65 Salgado, L. & Gasparini, Z. Reappraisal of an ankylosaurian dinosaur from the Upper Cretaceous of James Ross Island (Antarctica). *Geodiversitas* **28**, 119-135 (2006).

66 Lü, J., Jin, X., Sheng, Y. M. & Li, Y. H. New nodosaurid dinosaur from the late Cretaceous of Lishui, Zhejiang Province, China. *Acta Geol. Sin.* **81**, 344-350 (2007).

67 Boyd, C. A. The systematic relationships and biogeographic history of ornithischian dinosaurs. *PeerJ* **3**, e1523; 10.7717/peerj.1523 (2015).

68 Galton, P. M. Notes on Neocomian (Lower Cretaceous) ornithopod dinosaurs from England - *Hypsilophodon*, *valdosaurus*, “*Camptosaurus*”, “*Iguanodon*” - and referred specimens from Romania and elsewhere. *Rev. Paléobiol.* **28**, 211-273 (2009).

69 Seeley, H. G. On the dinosaurs from the Maastricht beds. *Q. J. Geol. Soc.* **39**, 246-253 (1883).

70 Ryan, M. J., Evans, D. C., Currie, P. J. & Loewen, M. A. A new chasmosaurine from northern Laramidia expands frill disparity in ceratopsid dinosaurs. *Naturwissenschaften* **101**, 505-512; 10.1007/s00114-014-1183-1 (2014).

71 Longrich, N. R. *Mojoceratops perifania*, a new chasmosaurine ceratopsid from the Late Campanian of western Canada. *J. Paleontol.* **84**, 681-694; 10.1666/09-114.1 (2010).

72 Farke, A. A. Anatomy and taxonomic status of the chasmosaurine ceratopsid *Nedoceratops hatcheri* from the Upper Cretaceous Lance Formation of Wyoming, USA. *PLOS One* **6**, e16196 (2011).

73 Schott, R. K. & Evans, D. C. Cranial variation and systematics of *Foraminacephale brevis* gen. nov. and the diversity of pachycephalosaurid dinosaurs (Ornithischia: Cerapoda) in the Belly River Group of Alberta, Canada. *Zool. J. Linn. Soc.*; 10.1111/zoj.12465 (2016).

74 Evans, D. C., Brown, C. M., You, H. L. & Campione, N. E. Description and revised diagnosis of Asia’s first pachycephalosaurid, *Sinocephale bexelli* gen. nov., from the Upper Cretaceous of inner Mongolia, China. *Can. J. Earth Sci.* **58**, 981-992 (2021).

75 Mehl, M. G. The Phytosauria of the Wyoming Triassic. *J. Denison Univ.* **28**, 141-172 (1928).

76 Hunt, A. P., Lucas, S. G. & Bircheff, P. Biochronological significance of the co-occurrence of the phytosaurs (Reptilia: Archosauria) *Angistorhinus* and *Rutiodon* in the Los Esteros Member of the Santa Rosa Formation, Santa Fe County, New Mexico, USA in *The nonmarine Triassic* 203-204 (New Mexico Museum of Natural History Bulletins, 1993)

77 Stocker, M. R. A new taxonomic arrangement for *Paleorhinus scurriensis*. *Earth Environ. Sci. Trans. R. Soc. Edinb.* **103**, 251-263; 10.1017/s1755691013000340 (2013).

78 Jones, A. S. & Butler, R. J. A new phylogenetic analysis of Phytosauria (Archosauria: Pseudosuchia) with the application of continuous and geometric morphometric character coding. *PeerJ* **6**, e5901; 10.7717/peerj.5901 (2018).

79 Griffin, C. T., Stefanic, C. M., Parker, W. G., Hungerbuhler, A. & Stocker, M. R. Sacral anatomy of the phytosaur *Smilosuchus adamanensis*, with implications for pelvic girdle evolution among Archosauriformes. *J. Anat.* **231**, 886-905; 10.1111/joa.12681 (2017).

80 Heckert, A. B. & Lucas, S. G. Stratigraphy and paleontology of the Lower Chinle Group (Adamanian: Latest Carnian) in the vicinity of St. Johns, Arizona. *N. M. Geol. Soc. Guidebook* **54**, 281-288 (2003).

81 Hunt, A. P., Lucas, S. G. & Spielmann, J. A. Sexual dimorphism in a large brachyrostral phytosaur (Archosauria: Crurotarsi) from the Late Triassic of western North America. *N. M. Mus. Nat. Hist. Sci. Bull.* **37**, 563-567 (2006).

82 Heckert, A. B., Lucas, S. G., Hunt, A. P. & Harris, J. D. A giant phytosaur (Reptilia: Archosauria) skull from the Redonda Formation (Upper Triassic: Apachean) of east-central New Mexico. *N. M. Geol. Soc. Guidebook* **52**, 171-178 (2001).

83 Huxley, T. H. The crocodilian remains found in the Elgin Sandstones: With remarks on the ichnites of Cummingstone. *Mem. Geol. Surv. U. K.* (1877).

84 Butler, R. J. *et al.* The sail-backed reptile *Ctenosauriscus* from the Latest Early Triassic of Germany and the timing and biogeography of the early archosaur radiation. *PLOS One* **6**, e25693 (2011).

85 Weinbaum, J. C. Postcranial skeleton of *Postosuchus kirkpatricki* (Archosauria: Paracrocodylomorpha), from the Upper Triassic of the United States. *Geol. Soc. Spec. Publ.* **379**, 525-553; 10.1144/sp379.7 (2013).

86 Melstrom, K. M. & Irmis, R. B. Repeated evolution of herbivorous crocodyliforms during the age of dinosaurs. *Curr. Biol.* **29**, 2389-2395 e2383; 10.1016/j.cub.2019.05.076 (2019).

87 Dollman, K. N., Clark, J. M., Viglietti, P. A., Browning, C. & Choiniere, J. N. Revised anatomy, taxonomy and biostratigraphy of *Notochampsa istedana* Broom, 1904, a Lower Jurassic crocodyliform from the Clarens Formation (Stormberg Group), and its implications for early crocodyliform phylogeny. *J. Syst. Palaeontol.* **19**, 651-675; 10.1080/14772019.2021.1948926 (2021).

88 Pol, D. & Powell, J. E. A new sebecid mesoeucrocodylian from the Rio Loro Formation (Palaeocene) of north-western Argentina. *Zool. J. Linn. Soc.* **163**, S7-S36; 10.1111/j.1096-3642.2011.00714.x (2011).

89 Souza, R. G. d., Hörmanseder, B. M., Figueiredo, R. G. & Campos, D. d. A. Description of new dyrosaurid specimens from the Late Cretaceous–Early Paleogene of New Jersey, United States, and comments on *Hyposaurus* systematics. *Hist. Biol.* **32**, 1377-1393; 10.1080/08912963.2019.1593403 (2019).

90 Halliday, T. A re-evaluation of goniopholidid crocodylomorph material from Central Asia: Biogeographic and phylogenetic implications. *Acta Palaeontol. Pol.*; 10.4202/app.2013.0018 (2013).

91 Efimov, M. B. Late Cretaceous crocodiles of Soviet Central Asia and Kazakhstan. *Paleontol. ž.* **9**, 417-420 (1975).

92 Buscalioni, A. D., Piras, P., Vullo, R., Signore, M. & Barbera, C. Early eusuchia crocodylomorpha from the vertebrate-rich Plattenkalk of Pietraroia (Lower Albian, southern Apennines, Italy). *Zool. J. Linn. Soc.* **163**, S199-S227; 10.1111/j.1096-3642.2011.00718.x (2011).

93 Buscalioni, Á. D., Alcalá, L., Espílez, E. & Mampel, L. European Goniopholididae from the Early Albian Escucha Formation in Ariño (Teruel, Aragón, Spain). *Span. J. Paleontol.* **28**, 103-122 (2013).

94 Kurzanov, S. Braincase structure in the carnosaur *Itemirus* n. gen. and some aspects of the cranial anatomy of dinosaurs. *Pap. Palaeontol.* **10**, 1-369 (1976).

95 Shuvalov, V. F. The Cretaceous stratigraphy and palaeobiology of Mongolia in *The age of dinosaurs in Russia and Mongolia* (ed. Benton, M. J. *et al.*) 256-278 2000)

96 Bona, P. & Barrios, F. The Alligatoroidea of Argentina: An update of its fossil record. *Publ. Electron. Asoc. Paleontol. Argent.*, 143-158; 10.5710/peapa.15.06.2015.103 (2015).

97 Müller, L. Ergebnisse der Forschungsreisen Prof. E. Stromers in den Wüsten Ägypteus in *Abhandlungen der Bayerischen Akademie der Wissenschaften. Mathematisch-Naturwissenschaftliche Abteilung* 1-97 (Oldenbourg Wissenschaftsverlag, 1927)

98 Marsh, O. C. Notice of some fossil mammals from the Tertiary Formation. *Am. J. Sci.* **2**, 35-44 (1871).
