# Supplementary material for: Deep reptilian evolutionary roots of a major avian respiratory adaptation
Source: Commun Biol. 2023 Jan 17;6:3. doi: 10.1038/s42003-022-04301-z (PMC9845227; doi:10.1038/s42003-022-04301-z)
Supplement: Supplementary file 3 — Description of Additional Supplementary Data [file 42003_2022_4301_MOESM3_ESM.pdf]

Description of Additional Supplementary Files

File name: Supplementary Data

Description: a .zip package containing: 1) raw data and script for the ancestral state reconstruction; 2) raw results generated from the ancestral state reconstruction; and 3) a Readme.txt file detailing the contents of the individual files within the .zip package.
