# Supplementary figures and images for: Deep reptilian evolutionary roots of a major avian respiratory adaptation
Source: Commun Biol. 2023 Jan 17;6:3. doi: 10.1038/s42003-022-04301-z (PMC9845227; doi:10.1038/s42003-022-04301-z)

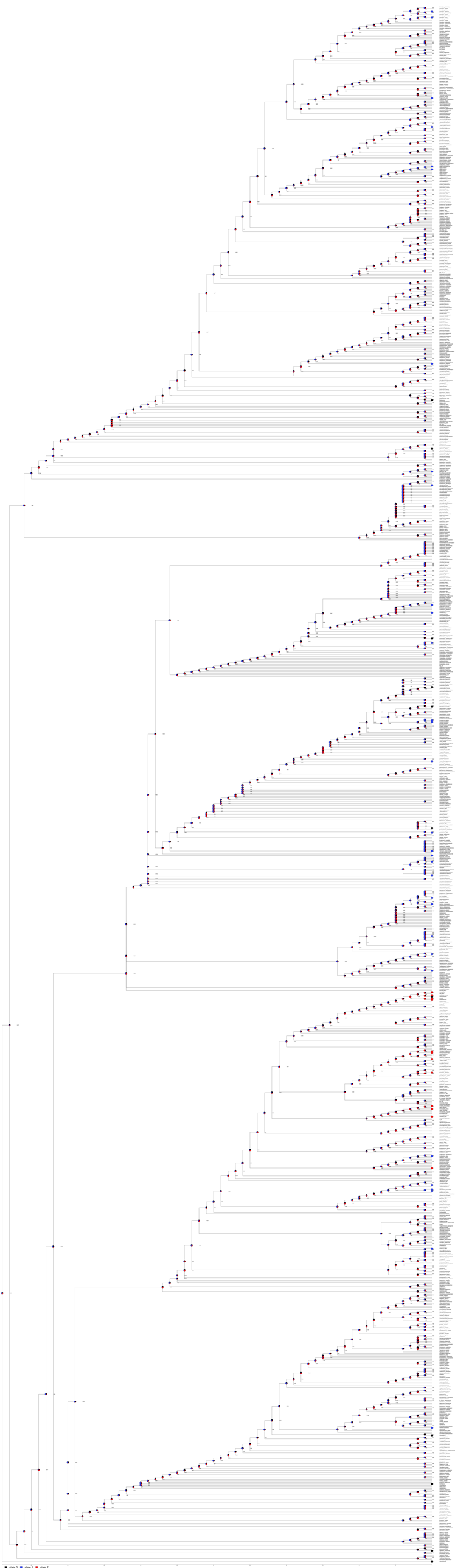

Supplement: Supplementary file 4 — Supplementary Data [file 42003_2022_4301_MOESM4_ESM.zip › Supplementary Data/ASR_results/Bayesian_ASR/MB_ac_br1.pdf]

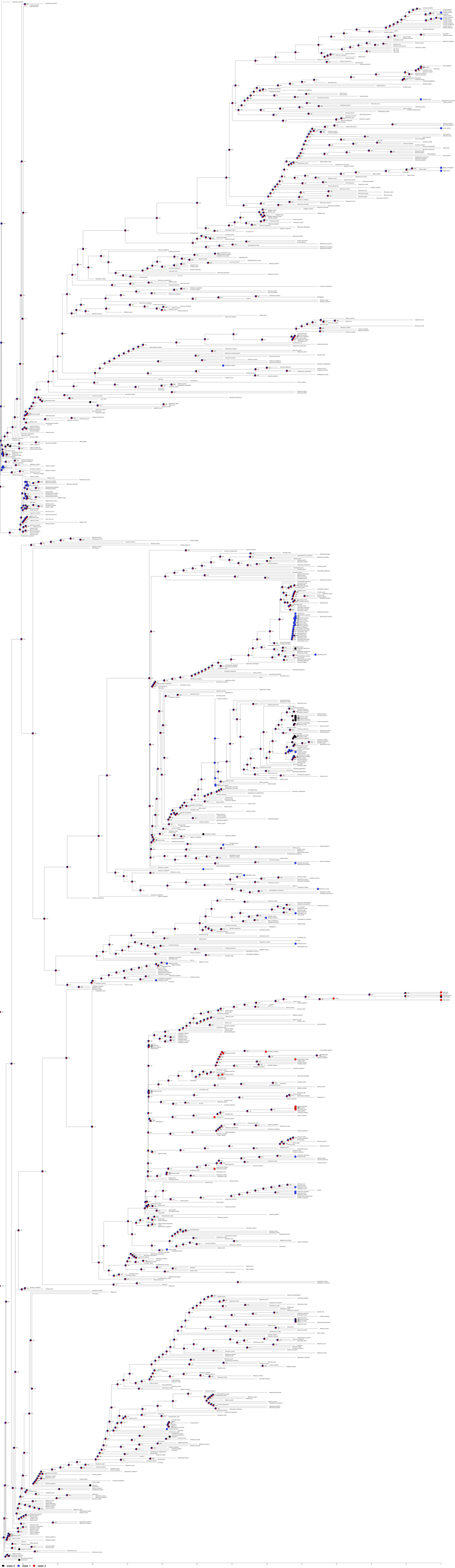

Supplement: Supplementary file 4 — Supplementary Data [file 42003_2022_4301_MOESM4_ESM.zip › Supplementary Data/ASR_results/Bayesian_ASR/MB_ac_ts.pdf]

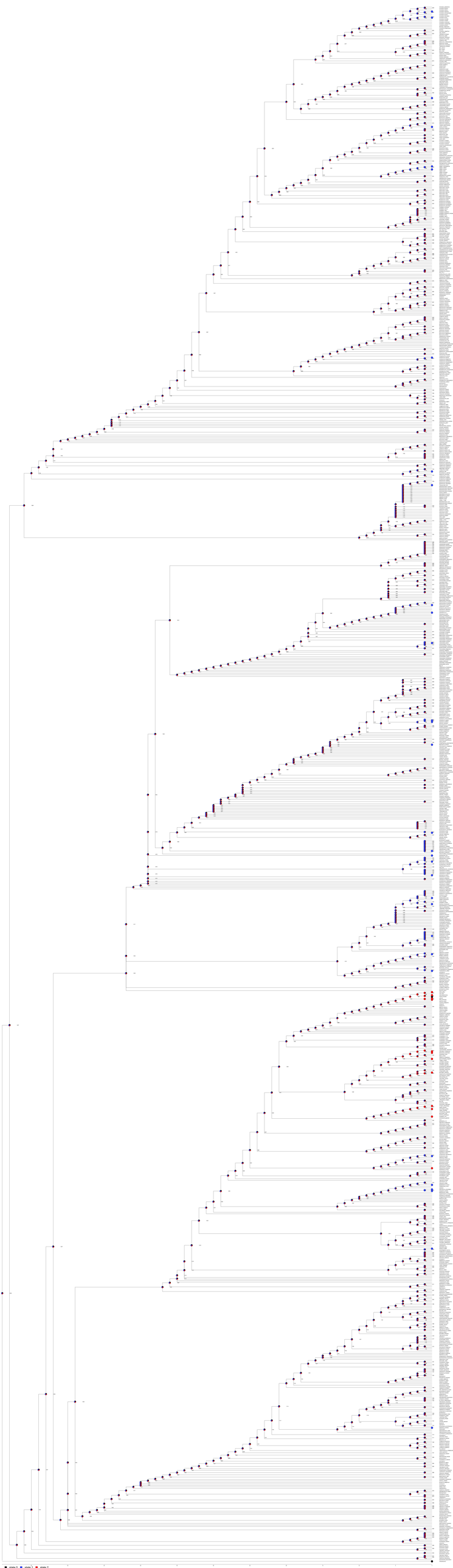

Supplement: Supplementary file 4 — Supplementary Data [file 42003_2022_4301_MOESM4_ESM.zip › Supplementary Data/ASR_results/Bayesian_ASR/MB_pc_br1.pdf]

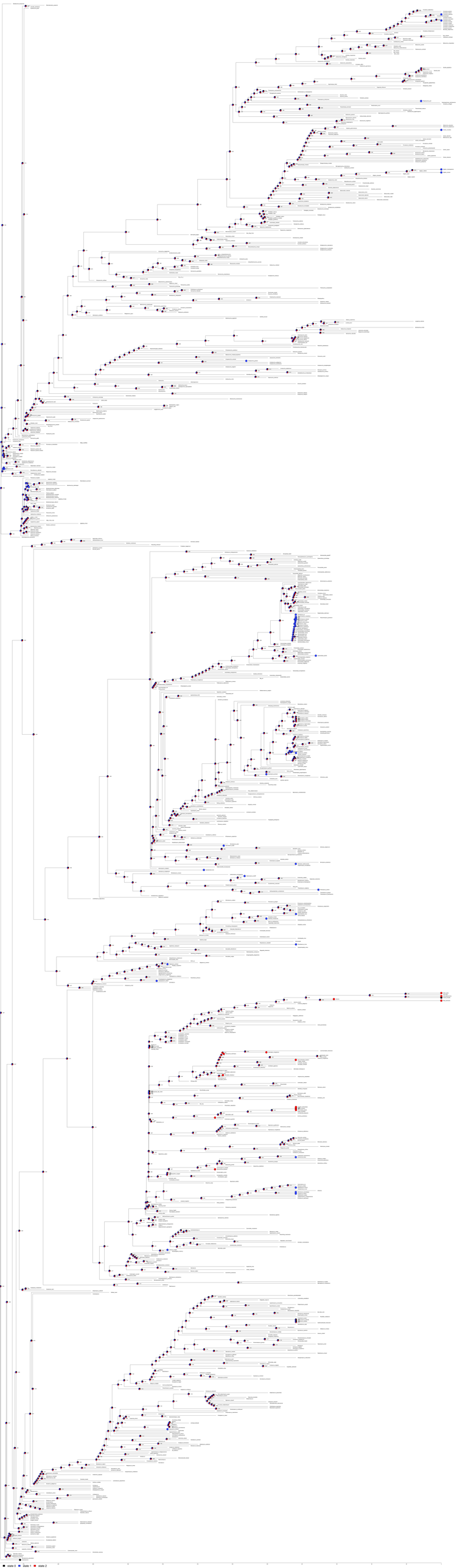

Supplement: Supplementary file 4 — Supplementary Data [file 42003_2022_4301_MOESM4_ESM.zip › Supplementary Data/ASR_results/Bayesian_ASR/MB_pc_ts.pdf]
