# Supplementary material for: Deep reptilian evolutionary roots of a major avian respiratory adaptation
Source: Commun Biol. 2023 Jan 17;6:3. doi: 10.1038/s42003-022-04301-z (PMC9845227; doi:10.1038/s42003-022-04301-z)
Supplement: Supplementary file 5 — Reporting Summary [file 42003_2022_4301_MOESM5_ESM.pdf]

nature portfolio

Corresponding author(s): Yan-yin Wang

Last updated by author(s): Nov 12, 2022

Reporting Summary

Nature Portfolio wishes to improve the reproducibility of the work that we publish. This form provides structure for consistency and transparency in reporting. For further information on Nature Portfolio policies, see our Editorial Policies and the Editorial Policy Checklist.

Statistics

For all statistical analyses, confirm that the following items are present in the figure legend, table legend, main text, or Methods section.

n/a	Confirmed	
☒	☐	The exact sample size (n) for each experimental group/condition, given as a discrete number and unit of measurement
☒	☐	A statement on whether measurements were taken from distinct samples or whether the same sample was measured repeatedly
☒	☐	The statistical test(s) used AND whether they are one- or two-sided<br>*Only common tests should be described solely by name; describe more complex techniques in the Methods section.*
☒	☐	A description of all covariates tested
☒	☐	A description of any assumptions or corrections, such as tests of normality and adjustment for multiple comparisons
☒	☐	A full description of the statistical parameters including central tendency (e.g. means) or other basic estimates (e.g. regression coefficient) AND variation (e.g. standard deviation) or associated estimates of uncertainty (e.g. confidence intervals)
☒	☐	For null hypothesis testing, the test statistic (e.g. F, t, r) with confidence intervals, effect sizes, degrees of freedom and P value noted<br>*Give P values as exact values whenever suitable.*
☒	☐	For Bayesian analysis, information on the choice of priors and Markov chain Monte Carlo settings
☒	☐	For hierarchical and complex designs, identification of the appropriate level for tests and full reporting of outcomes
☒	☐	Estimates of effect sizes (e.g. Cohen's d, Pearson's r), indicating how they were calculated

Our web collection on statistics for biologists contains articles on many of the points above.

Software and code

Policy information about availability of computer code

Data collection	No software was used to collect raw data; all raw data were collected via direct observation.
Data analysis	Ancestral state reconstruction was performed using R Studio 4.0. All raw data, and the script used to perform the analysis, are provided in the Supplementary Information.

For manuscripts utilizing custom algorithms or software that are central to the research but not yet described in published literature, software must be made available to editors and reviewers. We strongly encourage code deposition in a community repository (e.g. GitHub). See the Nature Portfolio guidelines for submitting code & software for further information.

Data

Policy information about availability of data

All manuscripts must include a data availability statement. This statement should provide the following information, where applicable:

- Accession codes, unique identifiers, or web links for publicly available datasets
- A description of any restrictions on data availability
- For clinical datasets or third party data, please ensure that the statement adheres to our policy

The authors declare that the data supporting the findings of this study are provided in the article and its Supplementary Data. Raw data and R scripts for the ancestral state reconstruction are provided in the Supplementary Data All specimens examined for this study are permanently housed in collections in Canada, China, and the United States. A full list of the examined specimens is provided in the Supplementary Information.

Human research participants

Policy information about studies involving human research participants and Sex and Gender in Research.

Reporting on sex and gender	*Use the terms sex (biological attribute) and gender (shaped by social and cultural circumstances) carefully in order to avoid confusing both terms. Indicate if findings apply to only one sex or gender; describe whether sex and gender were considered in study design whether sex and/or gender was determined based on self-reporting or assigned and methods used. Provide in the source data disaggregated sex and gender data where this information has been collected, and consent has been obtained for sharing of individual-level data; provide overall numbers in this Reporting Summary. Please state if this information has not been collected. Report sex- and gender-based analyses where performed, justify reasons for lack of sex- and gender-based analysis.*
Population characteristics	*Describe the covariate-relevant population characteristics of the human research participants (e.g. age, genotypic information, past and current diagnosis and treatment categories). If you filled out the behavioural & social sciences study design questions and have nothing to add here, write "See above."*
Recruitment	*Describe how participants were recruited. Outline any potential self-selection bias or other biases that may be present and how these are likely to impact results.*
Ethics oversight	*Identify the organization(s) that approved the study protocol.*

Note that full information on the approval of the study protocol must also be provided in the manuscript.

Field-specific reporting

Please select the one below that is the best fit for your research. If you are not sure, read the appropriate sections before making your selection.

☐ Life sciences ☐ Behavioural & social sciences ☒ Ecological, evolutionary & environmental sciences

For a reference copy of the document with all sections, see nature.com/documents/nr-reporting-summary-flat.pdf

Ecological, evolutionary & environmental sciences study design

All studies must disclose on these points even when the disclosure is negative.

Study description	Uncinate processes are ossified prongs or cartilaginous tabs extending from the posterior margins of the vertebral ribs in extant birds and crocodylians, respectively. Without being calcified, cartilages have low preservation potential in the fossil record, which makes identification of uncinate processes in deep time challenging. In this study, uncinate processes attachment sites were examined in extant birds and crocodylians, to establish osteological correlates for the presence of uncinate processes. The osteological correlate was then used to infer the presence of uncinate processes in extinct taxa, and the inferred distribution of uncinate processes was in turn used as a basis to determine if uncinate processes in modern birds and crocodylians may have been inherited from ancestral archosaurs.
Research sample	We examined dorsal vertebral ribs of extant bird and crocodylian housed at the Royal Ontario Museum and the University of Alberta Museum of Zoology. Where possible, uncinate processes were removed from the vertebral ribs to expose their attachment sites. We further examined vertebral ribs of fossil archosaurs housed in collections in Canada, China, and the United States (see Supplementary Information for details). Well-preserved and incomplete fossil vertebral ribs were both examined, though the former were prioritized. To augment these direct observations and provide more information for our ancestral state reconstruction, we obtained information from the literature on 17 fossil archosaurs with preserved uncinate processes, which in some cases are described as "intercostal plates".
Sampling strategy	We examined extant bird and crocodylian vertebral ribs in relatively small numbers, to establish the presence of an osteological correlate of uncinate process attachment in these groups, and as many well preserved vertebral ribs of extinct archosaurs as was logistically feasible. We searched the literature exhaustively for information on extinct archosaurs with preserved uncinate processes.
Data collection	Raw data were collected as qualitative descriptions and categorical character (e.g. presence of uncinate processes was coded as 1) based on direct observation of vertebral ribs, using a 10X hand lens and/or a Zeiss dissecting microscope with various magnification levels. Images of vertebral ribs showing osteological correlates of uncinate process attachment were captured using either a Zeiss camera attachment on the microscope, or a Canon 80D DSLR camera with a 100mm macro lens.
Timing and spatial scale	Most data were collected during research trips to visit museum collections: to China in November 2017, within Canada in May 2018, and to the United States in May and June 2019. Data from extant birds housed in the University of Alberta Museum of Zoology were collected periodically in 2018 and 2019.
Data exclusions	Taxonomically indeterminate specimens were excluded from the ancestral state reconstruction. The sole exceptions, an indeterminate phytosaur and an indeterminate aetosaur, were included as they were the only basal pseudosuchians with observed evidence of uncinate processes.
Reproducibility	Osteological correlates of uncinate processes attachment were documented and compared on multiple vertebral ribs for a given

Reproducibility	taxon if possible. High resolution images were captured, enabling others to verify/negate the authors' interpretations in the future. Alternate interpretations of the uncinate scars were discussed in the Supplementary Information. The ancestral state reconstruction was performed using an R script, which is provided in the Supplementary Data. A detailed description of the procedure used to perform the ancestral state reconstruction is also provided in the Supplementary Information.
Randomization	This study used a qualitative approach to describe and infer the distribution of uncinate processes across Archosauria. A randomization process was not required for this purpose.
Blinding	The subjects of this study were skeletal and cartilaginous elements of archosaurs, which did not have the ability to perceive information or provide responses. No blinding process was therefore considered necessary.

Did the study involve field work? ☐ Yes ☒ No

Reporting for specific materials, systems and methods

We require information from authors about some types of materials, experimental systems and methods used in many studies. Here, indicate whether each material, system or method listed is relevant to your study. If you are not sure if a list item applies to your research, read the appropriate section before selecting a response.

Materials & experimental systems

n/a	Involved in the study
☒	☐ Antibodies
☒	☐ Eukaryotic cell lines
☐	☒ Palaeontology and archaeology
☒	☐ Animals and other organisms
☒	☐ Clinical data
☒	☐ Dual use research of concern

Methods

n/a	Involved in the study
☒	☐ ChIP-seq
☒	☐ Flow cytometry
☒	☐ MRI-based neuroimaging

Palaeontology and Archaeology

Specimen provenance	No specimens were collected from the field in the course of this study. The specimens examined in this study are permanently housed in collections in Canada, China, and the United States.
Specimen deposition	Specimens can be accessed by visiting the American Museum of Natural History, New York, USA; Canadian Museum of Nature, Ottawa, Canada; Institute of Vertebrate Paleontology and Paleoanthropology, Beijing, China; Museum of Comparative Zoology, Cambridge, USA; New Mexico Museum of Natural History, Albuquerque, USA; Royal Ontario Museum, Toronto, Canada; Royal Tyrrell Museum of Palaeontology, Drumheller, Canada; University of Alberta Laboratory for Vertebrate Palaeontology, Edmonton, Canada; University of Alberta Museum of Zoology, Edmonton, Canada; and Yale Peabody Museum of Natural History, New Haven, USA.
Dating methods	No new geological dates are provided in this study.

☐ Tick this box to confirm that the raw and calibrated dates are available in the paper or in Supplementary Information.

Ethics oversight	No ethics approval was required for this study, because live human or animal subjects were not included in this study.

Note that full information on the approval of the study protocol must also be provided in the manuscript.
