# Supplementary material for: Deep reptilian evolutionary roots of a major avian respiratory adaptation
Source: Commun Biol. 2023 Jan 17;6:3. doi: 10.1038/s42003-022-04301-z (PMC9845227; doi:10.1038/s42003-022-04301-z)
Supplement: Supplementary file 4 — Supplementary Data [file MP_pc_ts.pdf › Supplementary Data/ASR_results/Maximum_parsimony_ASR/MP_pc_ts.pdf]

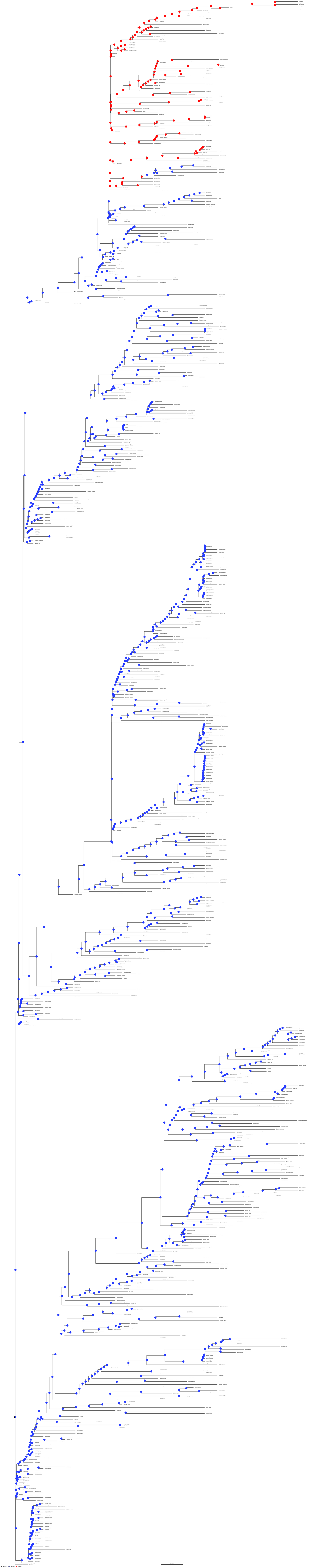

state 0
state 1
state 2
25 ma
